# Survival After Radical Cystectomy for Bladder Cancer: Development of a Fair Machine Learning Model

**DOI:** 10.2196/63289

**Published:** 2024-12-13

**Authors:** Samuel Carbunaru, Yassamin Neshatvar, Hyungrok Do, Katie Murray, Rajesh Ranganath, Madhur Nayan

**Affiliations:** 1 Department of Urology New York University School of Medicine New York, NY United States; 2 Department of Population Health New York University School of Medicine New York, NY United States; 3 Department of Urology Bellevue Hospital New York City Health and Hospitals New York, NY United States; 4 Center for Data Science New York University New York, NY United States; 5 Courant Institute of Mathematical Sciences New York University New York, NY United States

**Keywords:** machine learning, bladder cancer, survival, prediction, model, bias, fairness, radical cystectomy, mortality rate, algorithmic fairness, health equity, healthcare disparities

## Abstract

**Background:**

Prediction models based on machine learning (ML) methods are being increasingly developed and adopted in health care. However, these models may be prone to bias and considered unfair if they demonstrate variable performance in population subgroups. An unfair model is of particular concern in bladder cancer, where disparities have been identified in sex and racial subgroups.

**Objective:**

This study aims (1) to develop a ML model to predict survival after radical cystectomy for bladder cancer and evaluate for potential model bias in sex and racial subgroups; and (2) to compare algorithm unfairness mitigation techniques to improve model fairness.

**Methods:**

We trained and compared various ML classification algorithms to predict 5-year survival after radical cystectomy using the National Cancer Database. The primary model performance metric was the *F*_1_-score. The primary metric for model fairness was the equalized odds ratio (eOR). We compared 3 algorithm unfairness mitigation techniques to improve eOR.

**Results:**

We identified 16,481 patients; 23.1% (n=3800) were female, and 91.5% (n=15,080) were “White,” 5% (n=832) were “Black,” 2.3% (n=373) were “Hispanic,” and 1.2% (n=196) were “Asian.” The 5-year mortality rate was 75% (n=12,290). The best naive model was extreme gradient boosting (XGBoost), which had an *F*_1_-score of 0.860 and eOR of 0.619. All unfairness mitigation techniques increased the eOR, with correlation remover showing the highest increase and resulting in a final eOR of 0.750. This mitigated model had *F*_1_-scores of 0.86, 0.904, and 0.824 in the full, Black male, and Asian female test sets, respectively.

**Conclusions:**

The ML model predicting survival after radical cystectomy exhibited bias across sex and racial subgroups. By using algorithm unfairness mitigation techniques, we improved algorithmic fairness as measured by the eOR. Our study highlights the role of not only evaluating for model bias but also actively mitigating such disparities to ensure equitable health care delivery. We also deployed the first web-based fair ML model for predicting survival after radical cystectomy.

## Introduction

The development and use of prediction models is increasing in many domains, including health care. These models can be used to guide clinical decision-making as they can potentially provide more accurate predictions of outcomes by adjusting for multiple patient and clinical features, compared to clinician assessment alone [1,2]. Machine learning (ML) methods are increasingly being used to develop prediction models as their use often yields models with superior predictive performance compared to models developed using traditional statistical methods [3]. However, there have been growing concerns about the potential for bias in ML models, as studies have demonstrated discrepancies in model performance among different population subgroups, which can lead to disparities in health care [4-6]. This concern for model bias, or an unfair model, has brought attention to the concept of algorithm fairness, where a fair model is described as one that works well for all populations, regardless of personal characteristics that are considered protected or sensitive, such as sex, race, age, sexual orientation, among others [7,8].

An unfair model is of particular concern in bladder cancer, where disparities in the likelihood of receiving treatment and timing of treatment have been identified in sex and racial subgroups [9]. While several predictive models have been developed to predict survival in patients with bladder cancer [10-12], the performance of these models among different population subgroups remains understudied. Predicting survival is an important component in counseling patients with bladder cancer given the relatively high mortality rates in this disease [13,14]. The use of a potentially unfair model may perpetuate and even exacerbate existing disparities for vulnerable patient populations.

In this study, we used the National Cancer Database (NCDB) to develop a ML model that predicts survival after radical cystectomy, the current standard treatment for muscle-invasive bladder cancer [15], and evaluated for potential model unfairness in sex and racial subgroups. We then applied and compared model unfairness mitigation techniques to improve algorithm fairness [16] and deployed the first web-based fair ML model predicting survival after radical cystectomy.

## Methods

### Data Source

We used data from the NCDB, which captures approximately 70% of all new invasive cancer diagnoses in the United States each year. The NCDB is a joint project of the Commission on Cancer of the American College of Surgeons and the American Cancer Society. Reporting hospitals are restricted to those accredited by the American College of Surgeons Commission on Cancer. Data reporting to the NCDB is highly standardized, and all data submitted undergo a thorough review for verification of data integrity [17].

### Study Population

We identified patients with a histologic diagnosis of invasive urothelial carcinoma of the bladder between 2004 and 2016 and selected those who underwent radical cystectomy for clinically localized, muscle-invasive (cT2-4N0XM0X) disease. We excluded patients with missing dates of cystectomy; patients who received radiation, hormonal, immunotherapy, or other therapies prior to radical cystectomy; as well as patients who were treated with palliative intent.

For individuals included in our sample, we extracted patient and disease features. The patient features of interest were age at diagnosis, sex, race, ethnicity, facility type, insurance status, median income quartile, and comorbidity score; the disease factors were the year of diagnosis, pathological tumor stage, pathological nodal stage, and receipt of neoadjuvant or adjuvant chemotherapy.

We used the race and ethnicity features in the NCDB to categorize patients into the following National Institutes of Health (NIH) racial groups [18]: White, Black, Hispanic, or Asian. We a priori excluded American Indian or Alaska Native and Native Hawaiian or other Pacific Islanders as separate groups given their anticipated limited sample size [19,20]. The outcome of interest was overall survival at 5 years.

### Model Development and Evaluation

We used stratified splitting to separate the sample into training (13,184/16,481, 80%) and test (3297/16,481, 20%) sets, ensuring balance on sex, NIH racial group, and the outcome of interest.

We trained and compared various machine learning classification models, including random forest, decision tree, extreme gradient boosting (XGBoost), and logistic regression, to predict 5-year overall survival. We used 5-fold cross-validation to select the hyperparameters that optimized the *F*_1_-score. The baseline models were trained on the full training set, with optimized hyperparameters. We used a classification threshold of 0.5 for our baseline models.

Our primary model performance metric was the *F*_1_-score, which is the harmonic mean of the positive predictive value, also known as precision, and sensitivity, also known as recall; the formulas have been provided previously [21]. The *F*_1_-score can range from 0 to 1, with a value of 1 indicating perfect precision and recall. Secondary model performance metrics included true positive rate (TPR), false positive rate (FPR), and accuracy.

We evaluated model performance in the full test set and subgroups based on sex and race. The “best” naïve model was selected based on the highest *F*_1_-score in the full test set.

### Evaluation of Fairness

Several group fairness metrics have been described and fall under 3 broad principles: independence, separation, and sufficiency [8]. Independence is closely associated with demographic parity, which requires equivalent positive prediction rates across sensitive groups. Demographic parity is important when there are known historical biases in the dataset or objectivity of the target variable [8]. Separation is related to equalized odds and their relaxed variations; separation is suitable when the target variable is an objective ground truth and equality of error rates across sensitive groups is a priority. Equalized odds compare the TPR and FPR between different groups and are useful when true and false positives are considered to be of similar importance. Relaxed versions such as equality of opportunity and predictive equality can be used if prioritizing equal TPRs or FPRs, respectively. Separation-based criteria should not be used when the target variable may be prone to bias [8]. Finally, sufficiency is satisfied when for any predicted score, the probability of belonging to the positive or negative class is the same across all sensitive groups [8,22]. Unlike separation approaches, which may prioritize equalizing error rates across groups potentially at the expense of a specific group’s precision, sufficiency emphasizes fairness by ensuring similar prediction calibration for all groups without directly penalizing the overall model’s performance on any 1 group [22,23].

As some fairness criteria are not mutually compatible [24-26], for our clinical context, we used the equalized odds ratio (eOR) to estimate model fairness since we prioritized a model that minimized known disparities in bladder cancer by satisfying equality of error rates across sex and racial subgroups [8,27], there was no major concern for the retrospective data to contain measurement bias or historical bias, and true and false positive predictions of 5-year overall survival were of equal importance. The eOR ranges from 0 to 1, where a value of 1 indicates equivalent true positive and false positive rates across sensitive groups.

### Mitigating Unfairness

Techniques to mitigate algorithm unfairness can be applied in the 3 different phases of model development: preprocessing, in-processing, and postprocessing [8,28]. Techniques in the preprocessing category transform input data before they are passed to the training algorithm. For example, correlation remover is a preprocessing technique that applies a linear transformation to the nonsensitive features in order to remove any correlation with sensitive features. In practice, however, preprocessing approaches can still result in classifiers that result in substantial algorithm unfairness [29]. In-processing techniques incorporate fairness constraints within the model training process. These techniques aim to steer the model toward producing fair predictions. One such example is the exponentiated gradient method, which implements a reduction approach. In reduction approaches, the model is treated as a black box optimizer. The algorithm iteratively reweights the training data points based on the current model’s predictions and a chosen fairness metric. The model is then retrained with these reweighted points, aiming to reduce the unfairness observed in the previous iteration [29,30]. In postprocessing techniques, algorithms transform the output of a trained model. A threshold optimizer is an example of a postprocessing technique that takes as input an existing machine learning classifier and the sensitive feature, uses its predictions as a scoring function, and identifies a separate threshold for each group defined by a sensitive feature in order to optimize the specified fairness constraints [22]. Some limitations to note of the postprocessing approach are that they are not guaranteed to find the most accurate fair classifier and they require test time access to the protected attribute, which may not be available [29,31].

In this study, we compared 3 different techniques to mitigate unfairness in the different phases of model development: correlation remover (preprocessing), exponentiated gradient (in-processing), and threshold optimizer (postprocessing). The final “fair” model was chosen based on the mitigated model with the highest eOR in the full test set.

### Software and Analyses

All analyses were completed using Python (Python Software Foundation), and packages from *scikit-learn* and *Fairlearn* [16]. We estimated 95% CIs using 1000 bootstrap samples.

### Ethical Considerations

The study was exempted by our institutional review board committee since the NCDB does not contain any identifiable patient information.

## Results

### Population Characteristics

We identified 374,881 patients with a histologic diagnosis of invasive urothelial carcinoma of the bladder between 2004 and 2016 and selected those that underwent radical cystectomy for clinically localized, muscle-invasive (cT2-4N0XM0X) disease. After applying exclusion criteria, our overall final sample consisted of 16,481 patients, and their characteristics are shown in Table 1. The full sample was 23.1% (n=3800) female and 77% (n=12,681) male; with regard to the NIH racial groups, 91.5% (n=15,080), 5% (n=832), 2.3% (n=373), and 1.2% (n=196), were “White,” “Black,” “Hispanic,” and “Asian,” respectively (Multimedia Appendix 1). The median age at diagnosis in the overall sample was 71.0 (IQR 63.0-77.0) years, the majority of patients had pathological tumor stage 2 disease (n=7329, 44.5%; Table 1), and most patients were found to have negative pathological (pN0) lymph nodes (n=14,049, 85.2%).

Patient characteristics stratified by sex and race are shown in Table 2. Black male patients were the youngest (median age 66.5, IQR 58.0-74.0 y), on average, and had the highest proportion of pathological tumor stage 4 disease (116/512, 23%), while pathological lymph node involvement was highest in White male and Hispanic male patients (n=1599 and n=38 respectively, both 3.5%).

Of the 16,481 patients, there were 12,290 (75%) deaths within 5 years. Stratified by sex and race, death rates within 5 years were the highest in Black male patients (407/512, 80%) and the lowest in Asian male patients (101/148, 68.2%; Table 2).

**Table 1:** Baseline characteristics of patients undergoing radical cystectomy for bladder cancer.

Baseline characteristics		Full sample (N=16,481)	Training sample (n=13,184)	Test sample (n=3297)
**Age (years), median (IQR)**		71.0 (63.0-77.0)	71.0 (63.0-77.0)	71.0 (63.0-77.0)
**Sex, n (%)**				
	Female	3800 (23.1)	3039 (23.1)	761 (23.1)
	Male	12,681 (77)	10,145 (77)	2536 (77)
**NIH^a^** **racial group, n (%)**				
	Asian	196 (1.2)	157 (1.2)	39 (1.2)
	Black	832 (5)	666 (5)	166 (5)
	Hispanic	373 (2.3)	298 (2.3)	75 (2.3)
	White	15,080 (91.5)	12,063 (91.5)	3017 (91.5)
**Facility type, n (%)**				
	Community cancer program	785 (5)	628 (5)	157 (5)
	Comprehensive cancer program	5272 (32%)	4191 (32%)	1081 (33%)
	Academic or research program	7527 (46%)	6024 (46%)	1503 (46%)
	Integrated network program	2835 (17.2)	2294 (17.4)	541 (16.4)
	Unknown	62 (0.4)	47 (0.4)	15 (0.4)
**Insurance status, n (%)**				
	Private insurance	4568 (28)	3674 (28)	894 (27.1)
	Medicare	10,523 (64)	8414 (64)	2109 (64)
	Medicaid	616 (4)	470 (4)	146 (4.4)
	Other government	139 (1)	115 (1)	24 (0.7)
	Not insured	380 (2.3)	306 (2.3)	74 (2.2)
	Unknown	255 (2)	205 (2)	50 (1.5)
**Median income quartile (US $), n (%)**				
	<38,000	2403 (15)	1910 (14.5)	493 (15)
	38,000-47,999	3800 (23.1)	3004 (23)	796 (24.1)
	48,000 - 62,999	4135 (25.1)	3327 (25.2)	808 (24.5)
	≥63,000	4587 (28)	3699 (28.1)	888 (27)
	Unknown	1556 (9.4)	1244 (9.4)	312 (10)
**Charlson-Deyo comorbidity score, n (%)**				
	0	10,901 (66.1)	8693 (66)	2208 (67)
	1	3950 (24)	3167 (24)	783 (24)
	2	1222 (7.4)	1000 (7.58)	222 (6.73)
	3	408 (2.48)	324 (2.46)	84 (2.55)
**Year of diagnosis, median (IQR)**		2009 (2007-2012)	2010 (2007-2012)	2009 (2007-2012)
**Pathological tumor stage, n (%)**				
	pT2	7329 (44.5)	5869 (44.5)	1460 (44.3)
	pT3	6960 (42.2)	5569 (42.2)	1391 (42.3)
	pT4	2192 (13.3)	1746 (13.2)	446 (13.5)
**Pathological nodal stage, n (%)**				
	pNX	1971 (12)	1612 (12)	359 (11)
	pN0	14,049 (85.2)	11,196 (85)	2853 (86.5)
	pN+	461 (3)	376 (3)	85 (3)
**Receipt of chemotherapy, n (%)**				
	Neoadjuvant only	1501 (9.1)	1213 (9.2)	288 (9)
	Adjuvant only	1292 (8)	1049 (8)	243 (7.4)
	Both neoadjuvant and adjuvant	13,688 (83.1)	10,922 (83)	2766 (84)

^a^NIH: National Institutes of Health.

**Table 2:** Baseline characteristics of patients undergoing radical cystectomy for bladder cancer, stratified by sex and race.

Baseline characteristics		White female patients (n=3342)	White male patients (n=11738)	Black female patients (n=320)	Black male patients (n=512)	Asian female patients (n=48)	Asian male patients (n=148)	Hispanic female patients (n=90)	Hispanic male patients (n=283)
**Age (years), median (IQR)**		72.0 (64.0-78.0)	70.0 (63.0-77.0)	67.0 (58.0-74.0)	66.5 (58.0-74.0)	75.0 (64.0-79.0)	71.0 (62.0-77.3)	73 (66.0-77.0)	70 (63-75)
**Facility type, n (%)**									
	Community cancer program	168 (5)	565 (5)	8 (2.5)	19 (4)	4 (8.3)	5 (3.4)	5 (6)	11 (4)
	Comprehensive community cancer program	1125 (34)	3819 (32.5)	89 (28)	113 (22.1)	13 (27.1)	30 (20.3)	15 (17)	68 (24)
	Academic programs	1422 (42.5)	5328 (45.4)	164 (51.3)	279 (54.5)	23 (48%)	89 (60.1)	55 (61.1)	167 (59)
	Integrated network cancer program	605 (18.1)	1996 (17)	56 (17.5)	99 (19.3)	8 (17)	24 (16.2)	12 (13.3)	35 (12.4)
	Unknown	22 (1)	30 (0.3)	3 (1)	2 (0.4)	0 (0)	0 (0)	3 (3.3)	2 (1)
**Insurance status, n (%)**									
	Private insurance	836 (25)	3343 (28.5)	83 (26)	152 (30)	14 (29.2)	46 (31.1)	20 (22.2)	74 (26.1)
	Medicare	2263 (68)	7447 (63.4)	190 (59.4)	285 (56)	26 (54.2)	79 (53.4)	59 (66)	174 (61.5)
	Medicaid	116 (3.5)	382 (3.2)	31 (10)	45 (9)	4 (8.3)	16 (11)	8 (9)	14 (5)
	Other government	12 (0.4)	117 (0.1)	2 (1)	5 (1)	1 (2.1)	6 (4)	1 (1.1)	1 (0.4)
	Not insured	66 (2)	262 (2.2)	11 (3.4)	19 (4)	2 (4.2)	1 (1)	2 (2.2)	12 (4.2)
	Unknown	49 (1.5)	187 (2)	3 (1)	6 (1.2)	1 (2.1)	0 (0)	0 (0)	8 (3)
**Median income quartile (US $),** **n (%)**									
	<38,000	447 (13.4)	1550 (13.2)	113 (35.3)	194 (38)	2 (4.2)	5 (3.4)	21 (23.3)	71 (25.1)
	38,000-47,999	820 (24.5)	2692 (23)	64 (20)	110 (21.5)	3 (6.2)	24 (16.2)	20 (22.2)	67 (24)
	48,000-62,999	830 (25)	3014 (26)	54 (17)	95 (19)	13 (27.1)	38 (26)	22 (24.4)	69 (24.4)
	≥63,000	929 (28)	3351 (28.5)	51 (16)	73 (14.3)	28 (58.3)	71 (48)	24 (27)	60 (21.2)
	Unknown	316 (9.5)	1131 (10)	38 (12)	40 (8)	2 (4.2)	10 (7)	3 (3.3)	16 (6)
**Comorbidity score,** **(n (%)**									
	0	2292 (69)	7648 (65.2)	201 (63)	375 (73.2)	39 (81.3)	97 (65.5)	57 (63.3)	192 (68)
	1	767 (23)	2880 (24.5)	80 (25)	89 (17.4)	8 (17)	42 (28.4)	20 (22.2)	64 (23)
	2	213 (6.4)	910 (8%)	34 (11)	33 (6.4)	1 (2.1)	7 (5)	8 (9%)	16 (6)
	≥3	70 (2.1)	300 (3)	5 (2)	15 (3)	0 (0)	2 (1.4)	5 (6)	11 (4)
**Year of diagnosis, median (IQR)**		2009 (2006-2012)	2009 (2007-2012)	2010 (2008-2013)	2010 (2007-2013)	2010 (2007-2013)	2009 (2007-2012)	2009 (2007-2013	2010 (2006-2013)
**Pathological tumor** **stage, n (%)**									
	pT2	1450 (43.4)	5290 (45.1)	141 (44.1)	213 (42)	18 (37.5)	60 (40.5)	26 (29)	131 (46.3)
	pT3	1534 (46)	4849 (41.3)	145 (45.3)	183 (36)	20 (42)	68 (46)	47 (52.2)	114 (40.3)
	pT4	358 (11)	1599 (14)	34 (11)	116 (23)	10 (21)	20 (13.5)	17 (19)	38 (13.4)
**Pathological nodal stage,** (n (%)									
	pNX	431 (13)	1353 (11.5)	40 (12.5)	73 (14.3)	4 (8.33)	16 (11)	10 (11.1)	44 (15.5)
	pN0	2793 (84)	10081 (86)	271 (85)	423 (83)	44 (92)	130 (88)	78 (87)	229 (81)
	pN+	118 (3.5)	304 (3)	9 (3)	16 (3.1)	0 (0)	2 (1.4)	2 (2.2)	10 (3.5)
**Receipt of chemotherapy,** **(n (%)**									
	Neoadjuvant	317 (9.5)	1056 (9)	41 (13)	43 (8.4)	0 (0)	8 (5.4)	12 (13.3)	24 (8.5)
	Adjuvant	263 (8)	930 (8)	19 (6)	35 (7)	3 (6.2)	7 (5)	14 (16)	21 (7.4)
	Neither	2762 (83)	9752 (83.1)	260 (81.3)	434 (85)	45 (94)	133 (90)	64 (71.1)	238 (84.1)
**Death within 5 years, n (%)**		2437 (73)	8790 (75)	240 (75)	407 (80)	34 (71)	101 (68.2)	62 (69)	219 (77.4)

### Naïve Model Performance and Evaluation of Fairness

The performance of the naïve models is shown in Table 3. The “best” naïve model based on *F*_1_-score was XGBoost (*F*_1_-score 0.860; 95% CI 0.8490.869). When evaluated for fairness, the “best” naïve model had an eOR ratio of 0.619 (Table 4).

**Figure 1:**
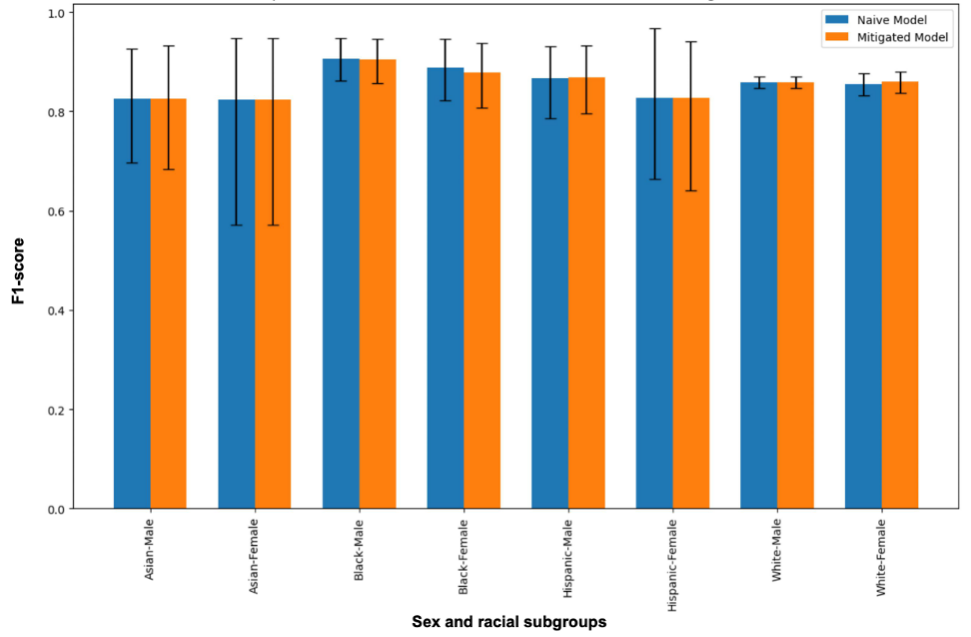
Comparison of F1-scores between best naïve and mitigated models, in sex and racial subgroups.

In sex and racial subgroups, the “best” naïve model achieved the highest *F*_1_-score in Black male patients (*F*_1_-score 0.907; 95% CI 0.859-0.947) and the lowest in Asian female patients (*F*_1_-score 0.824; 95% CI 0.571-0.947; Figure 1).

**Table 3:** Comparison of the naïve machine learning models predicting 5-year survival in patients undergoing radical cystectomy for bladder cancer.

	*F*_1_-score	True positive rate	False positive rate	Accuracy
XGBoost^a^	0.860 (0.849-0.869)	0.970 (0.960-0.973)	0.830 (0.803-0.869)	0.765 (0.749-0.778)
Random forest	0.832 (0.820-0.842)	0.854 (0.841-0.867)	0.585 (0.552-0.621)	0.742 (0.728-0.757)
Decision tree	0.719 (0.701-0.734)	0.632 (0.611-0.649)	0.367 (0.336-0.400)	0.632 (0.616-0.647)
Logistic regression	0.852 (0.841-0.862)	0.9378 (0.928-0.946)	0.775 (0.745-0.803)	0.756 (0.742-0.771)

^a^XGBoost: extreme gradient boosting.

**Table 4:** Comparison of the best naive and mitigated models to predict 5-year survival in patients undergoing radical cystectomy for bladder cancer.

	Equalized odds ratio	*F*_1_-score	True positive rate	False positive rate	Accuracy
Naive model	0.619	0.860 (0.849-0.869)	0.970 (0.960-0.973)	0.830 (0.803-0.869)	0.765 (0.749-0.778)
Correlation remover	0.750	0.861 (0.851-0.870)	0.966 (0.959-0.973)	0.819 (0.792-0.843)	0.766 (0.751-0.787)
Exponentiated gradient	0.667	0.861 (0.851-0.870)	0.963 (0.956-0.971)	0.807 (0.780-0.834)	0.767 (0.752-0.781)
Threshold optimizer	0.750	0.847 (0.837-0.857)	0.958 (0.949-0.965)	0.889 (0.868-0.910)	0.742 (0.727-0.757)

### Mitigating Unfairness

We compared the preprocessing, in-processing, and postprocessing algorithm unfairness mitigation techniques of correlation remover, exponentiated gradient, and threshold optimizer, respectively. We found that all techniques improved the eOR, with the largest improvements observed after applying correlation remover and threshold optimizer; application of these techniques resulted in an equivalent eOR of 0.750 (Table 4). The mitigated model using correlation remover had a higher *F*_1_-score and was selected as our final “fair” model (Table 4).

The comparison of the “best” naïve and “fair” final models in sex and racial subgroups is shown in Figure 1. We found that compared to the “best” naïve model, the *F*_1_-score of the “fair” final model did not change significantly for any of the subgroup populations. Secondary performance metrics in subgroups are shown in Multimedia Appendix 2.

## Discussion

In this study, we used the NCDB to develop a ML model predicting survival after radical cystectomy for bladder cancer and found that the naïve model would be considered unfair due to inferior performance in certain sex and racial subgroups. We then compared and used algorithm unfairness mitigation techniques in the 3 different phases of model development and found that all techniques improved model fairness, with negligible impact on overall classifier accuracy. Our study highlights the importance of evaluating prediction models in health care for potential unfairness and demonstrates different techniques that can be applied to mitigate model unfairness. We also deploy the first web-based “fair” model predicting survival in patients undergoing radical cystectomy for muscle-invasive bladder cancer.

The potential consequences of algorithm unfairness have been highlighted in previous studies. In a study that evaluated software used by courts in the United States to decide whether to release an offender or to keep them in prison, they found higher FPRs for Black offenders, compared to White offenders [6]. The bias in this model is partly due to the skewed training data in which the 2 populations had unequal base rates of being charged with new crimes [32]. These discrepant base rates may be partially attributable to heavier policing in predominantly Black neighborhoods or bias in the decision to make an arrest [33]. Another study evaluated a model used by health insurers to select patients for complex care management programs and found that for the same level of model-predicted risk, Black patients have significantly higher illness burdens, compared to White patients [5]. This bias was attributed to the algorithm’s objective of accurately predicting costs, which biases against Black patients who have been shown to generate lower medical expenses, conditional on health. A review of clinical ML algorithms found that the majority of models exhibited some form of racial bias [28]. The application of these unfair models could amplify and perpetuate disparities for vulnerable populations. Fairness methods can be used to mitigate known biases; in the context of the prison software, for example, historical biases in the data could be addressed by developing a model to optimize demographic parity [8]. In the context of the biased complex care algorithm, fairness would be improved by having the model’s objective changed to accurately predict active chronic conditions, rather than costs [5].

In bladder cancer, several prediction models have been developed to predict survival [10-12], although none have been evaluated for potential unfairness in sex and racial subgroups. In this study, we found that a model predicting survival after radical cystectomy demonstrated unfairness whereby specific subgroups, such as Black male patients, had superior model performance compared to others, such as Asian female patients. If such an algorithm was used to support decision-making, quality-of-service harm may result in subgroups with less accurate predictions [31].

Identifying the source of model unfairness can be challenging, and several potential causes have been described [7,8,28,31]. Sampling bias occurs when the model performs worse in minority populations due to insufficient sampling during training [7]. This source of bias is of potential concern when developing prediction models in diseases such as bladder cancer, where the epidemiology is significantly skewed by sex and race; worldwide, bladder cancer is approximately 4 times more common in male patients than female patients [34] and in the United States, bladder cancer is approximately 2 times more common in White populations compared to Black, Hispanic, and Asian populations [35]. Given this skewed demographic, which was reflected in our study, a naïve ML model may not perform equally well in all subgroups of patients. However, despite Black male patients representing only 3.1% of our sample, we found that the naïve model performed best in this subgroup. Notably, Black male patients had the highest mortality rate at 5 years, and this imbalanced data may partly explain the superior model performance in this subgroup. This aligns with prior research where a ML model predicting survival in patients with prostate cancer undergoing radical prostatectomy showed superior performance in Black patients, which was also the minority group with the highest mortality rate in that study [4].

While the potential for model bias has been described in previous studies, research on applying techniques to mitigate algorithmic unfairness remains limited [28]. However, a recent scoping review found that all studies implementing methods to address racial bias successfully improved fairness metrics [28]. This underscores the value of using such techniques before model deployment to optimize fairness. Beyond metrics, ensuring that a model is perceived as fair is particularly important in health care as it can enhance trust among both patients and clinicians, potentially leading to greater acceptance and integration of the model’s predictions into clinical decision-making. Our study also highlights the importance of evaluating and comparing multiple techniques to achieve optimal fairness, as there was variability in the degree of improvement observed between each approach.

Our study is not without limitations. Despite the use of model unfairness techniques which improved the eORs of the naïve model, the final “fair” model did not achieve an eOR of 1, indicating the presence of residual unfairness; however, some might argue that an eOR of 1 is theoretical and could not be achieved in practice. This concept was illustrated in a study that demonstrated the restrictive conditions required for a deterministic classifier to achieve perfect equalized odds [36]. Therefore, while approaches can be used to reduce algorithmic unfairness, clinicians and patients should still be aware of residual potential discrepancies in model performance. Another limitation of optimizing eOR is that it violates other definitions of fairness and may compromise performance in certain subgroups [26]. In our study, we found that while the *F*_1_-score of the mitigated model improved or was unchanged in some subgroups, performance decreased in certain subgroups, highlighting the potential trade-off between fairness and performance. This raises an ethically complex question regarding what is considered “fair,” and the answer varies depending on what conceptual framework of fairness is used. We specifically focused on the pillar of inclusion which seeks to ensure that the benefits and harms from prediction models are distributed in an equitable manner [37]. The eOR is also challenged as a metric in the context of imbalanced data, as is common in health care, as baseline differences can contribute to disparities in performance. However, despite the expected differences across sex and racial subgroups in our study, we prioritized the eOR to develop a model that satisfied equality of error rates across subgroups [8,27] and where true and false positive predictions of 5-year overall survival were of equal importance. Finally, our study uses NIH racial groups, which are composed of heterogeneous subgroups; we did not evaluate model performance in these subgroups given their expected limited sample size. Additionally, we chose to focus on sex and race given the existing disparities in bladder cancer among these subgroups [9], but other sensitive subgroups, such as those based on socioeconomic status or rurality, could be studied for potential bias in model performance. Despite these limitations, our study demonstrates the importance of evaluating model unfairness and, to the best of our knowledge, is the first to apply unfairness mitigation techniques to reduce disparities from potential algorithmic unfairness in bladder cancer.

In conclusion, we developed a ML model to predict survival after radical cystectomy for bladder cancer and found that a naïve model exhibited bias, as certain subgroups of patients, based on sex and race, had inferior performance compared to others. Using algorithm unfairness mitigation techniques to minimize potential disparities arising from biased models, we improved model fairness and deployed the first web-based fair ML model for predicting survival after radical cystectomy.

## Appendix

Multimedia Appendix 1 Distribution of sample by sex and race of patients undergoing radical cystectomy for bladder cancer.

Multimedia Appendix 2 Comparison of best naïve and fair models in sex and race subgroups.

## Abbreviations

eOR: equalized odds ratio
FPR: false positive rate
ML: machine learning
NCDB: National Cancer Database
NIH: National Institutes of Health
TPR: true positive rate
XGBoost: extreme gradient boosting
