# Supplementary figures and images for: Survival After Radical Cystectomy for Bladder Cancer: Development of a Fair Machine Learning Model
Source: JMIR Med Inform. 2024 Dec 13;12:e63289. doi: 10.2196/63289 (PMC11694706; doi:10.2196/63289)

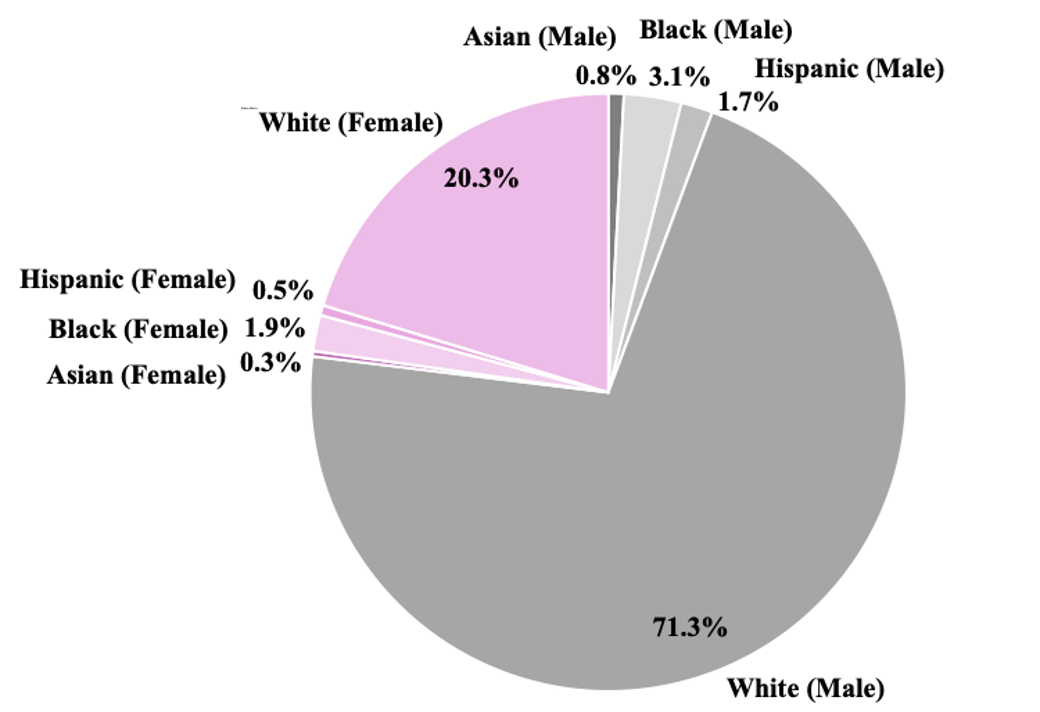

Supplement: Multimedia Appendix 1 [file medinform_v12i1e63289_app1.png]
