# Supplementary material for: Survival After Radical Cystectomy for Bladder Cancer: Development of a Fair Machine Learning Model
Source: JMIR Med Inform. 2024 Dec 13;12:e63289. doi: 10.2196/63289 (PMC11694706; doi:10.2196/63289)
Supplement: Multimedia Appendix 2 [file medinform_v12i1e63289_app2.docx]

Multimedia Appendix 2: Comparison of best naïve and fair models in sex and race subgroups.

|  | **F1 score** | **True Positive Rate** | **False Positive Rate** | **Accuracy** |
| --- | --- | --- | --- | --- |
| Black males  naive model | 0.907  (0.859-0.947) | 0.963  (0.916-1.0) | 0.619  (0.400-0.815) | 0.843  (0.765-0.912) |
| Black males  mitigated model | 0.895  (0.850-0.940) | 0.951  (0.902-0.988) | 0.667  (0.450-0.857) | 0.824  (0.745-0.892) |
| Black females naive model | 0.889  (0.816-0.944) | 1.0  (1.0-1.0) | 0.75  (0.526-0.944) | 0.8125  (0.719-0.906) |
| Black females mitigated model | 0.862  (0.792-0.930) | 0.979  (0.933-1.0) | 0.875  (0.667-1.0) | 0.766  (0.656-0.875) |
| White males  naive model | 0.859  (0.848-0.870) | 0.964  (0.954-0.972) | 0.836  (0.805-0.866) | 0.7628  (0.746-0.782) |
| White males  mitigated model | 0.860  (0.848-0.871) | 0.960  (0.951-0.969) | 0.812  (0.779-0.841) | 0.767  (0.750-0.783) |
| White females naive model | 0.855  (0.830-0.876) | 0.977  (0.963-0.988) | 0.834  (0.781-0.885) | 0.758  (0.728, 0.788) |
| White females mitigated model | 0.858  (0.837-0.88) | 0.973  (0.958-0.986) | 0.7956  (0.735-0.852) | 0.765  (0.734-0.795) |
| Asian males  naive model | 0.826  (0.698-0.926) | 0.950  (0.850-1.0) | 0.778  (0.444-1.0) | 0.724  (0.552-0.897) |
| Asian males  mitigated model | 0.826  (0.683-0.936) | 0.95  (0.833-1.0) | 0.778  (0.428-1.0) | 0.724  (0.586-0.897) |
| Asian females naive model | 0.824  (0.571-0.947) | 1.0  (1.0-1.0) | 1.0  (1.0-1.0) | 0.700   (0.400-0.900) |
| Asian females mitigated model | 0.824  (0.571-0.947) | 1.0   (1.0-1.0) | 1.0  (1.0-1.0) | 0.700  (0.400-0.900) |
| Hispanic males  naive model | 0.866  (0.791-0.926) | 0.955  (0.886-1.0) | 0.846  (0.643-1.0) | 0.772  (0.667-0.877) |
| Hispanic males  mitigated model | 0.866  (0.791-0.932) | 0.955  (0.886-1.0) | 0.846  (0.611-1.0) | 0.772  (0.667-0.877) |
| Hispanic females naive model | 0.828  (0.640-0.941) | 1.0  (1.0-1.0) | 0.833  (0.500-1.0) | 0.722  (0.500-0.944) |
| Hispanic females mitigated model | 0.828  (0.667-0.966) | 1.0  (1.0-1.0) | 0.833  (0.500-1.0) | 0.722   (0.500-0.889) |
